# Supplementary material for: Gender differences in anxiety and perceived stress during the Covid-19 pandemic among Brazilian adolescents from impoverished communities
Source: PLOS Ment Health. 2025 Aug 18;2(8):e0000380. doi: 10.1371/journal.pmen.0000380 (PMC12798566; doi:10.1371/journal.pmen.0000380)
Supplement: Attachment — Submitted filename: Response to Reviewers.docx [file pmen.0000380.s003.docx]

July 1^st^, 2025

To the Editors-in-Chief,

Charlene Sunkel and Rochelle Burgess,

PLOS Mental Health

**Ref:** Gender differences in anxiety and perceived stress during the Covid-19 pandemic among Brazilian adolescents from impoverished communities

**ID:** PMEN-D-25-00090

Dear Editors,

We would like to express our sincere appreciation for the reviewers’ thoughtful evaluation of our manuscript. The insightful comments and constructive suggestions provided to us have played a pivotal role in improving both the quality and clarity of our work. In response, we carefully examined each point raised and implemented the necessary revisions, which we believe effectively address the concerns presented. Detailed responses to all comments are outlined below, and the corresponding changes have been highlighted in yellow in the revised version of the manuscript.

In addition to addressing the reviewers’ observations, we took the opportunity to implement a series of minor corrections throughout the manuscript, all of which have been also marked in yellow. These adjustments included the insertion of new references and the removal of others that do not correspond to the actual citations in the main text. Such modifications altered the numbering of the references, leading to a renumbering of most citations throughout the manuscript. We also added brief transitional phrases to improve textual cohesion and made several other minor refinements. Finally, the abstract was rewritten to more accurately reflect the revised structure and principal results of the study.

We trust that the revised manuscript now meets the editorial expectations and review criteria, and we remain fully open to any further suggestions, clarifications, or corrections that may contribute to the continued improvement of our submission.

Sincerely,

The Authors

**Reviewer #1:**

Dear Reviewer 1,

Thank you very much for your thoughtful comments. Please find our detailed responses to each of your points below.

1. There is a point that I would like to discuss that refers to a hypothesis presented by the authors in light of the result that girls have greater experiences of anxiety and perceived stress than boys. According to the authors’ hypothesis, the difference between genders refers to behavioral norms, impregnated by machismo, socially imposed, which make boys repress their emotional expressions, unlike girls who are encouraged to show their emotions more abundantly. I do not disagree with this interpretation, but I believe that another interpretation is also possible. Women and girls, in general, in a society marked by patriarchy as an important social marker in Western society and in Brazil, a colonized society, receive a stricter education and have their behavior more controlled than men and boys. In other words, in a context of domestic confinement, the fear of suffering repression or insults from adults is much more frequent for girls than for boys, who are raised with much greater freedom of behavior. Girls are more vulnerable to domestic violence than boys. And the variable fear of being insulted or not receiving love from adults in the house may reflect situations of domestic violence, even if not physical, but possible to be psychologically applicable. It is suggested that the authors also reflect on the issue with scientific literature that shows the rates of domestic violence and gender-based violence experienced during the COVID-19 pandemic.

**Authors’ response:** We thank the reviewer for the thoughtful and analytically rich observation, which substantially enriches the interpretive depth of our findings. The suggestion to consider not only the expressive dimensions of gendered emotional norms but also the structural constraints to which girls are disproportionately subjected, particularly within contexts marked by patriarchy and the enduring legacies of colonialism (as is the case in Brazil), prompted important revisions to our manuscript.

In response, we expanded both the Introduction and Discussion sections in order to move beyond the reductive dichotomy between emotional expressivity and repression. The revised text now incorporates the interplay between emotional vulnerability and intensified moral surveillance, behavioral control, and symbolic or psychological violence, which often characterize girls’ experiences in domestic environments. This broadened perspective allows for a more comprehensive understanding of the emotional distress reported, particularly under conditions of confinement such as those imposed by the Covid-19 pandemic.

To set the stage for this expanded discussion and to prevent a reductive interpretation of our initial hypothesis, we included a reference to this idea in the Introduction section (pages 3-4, lines 75-83). In this passage, we emphasize that gender norms, although embodied in distinct ways, shape the emotional development of boys and girls in interdependent yet asymmetrical patterns.

The newly added paragraphs in the Discussion (pages 30-31, lines 388-410; pages 35-36, lines 504-534) articulate several key points: (1) the normative pressures that regulate girls’ emotional expression through intensified domestic oversight; (2) the consequences of growing up under stricter behavioral control and heightened moral expectations; and (3) international and national empirical evidence documenting the increase in gender-based and domestic violence during the pandemic, which underscores the systemic vulnerabilities to which girls remain disproportionately exposed. These points were further reinforced in the Conclusion (page 37, lines 555-562).

By integrating these complementary interpretative strands, we sought to offer a more nuanced and theoretically grounded reading of the observed gender differences. This framing situates our findings within broader social and historical logics that regulate the visibility, legitimacy, and articulation of emotional suffering.

2. Another point that I consider important is the issue related to race/skin color. Although the race/color factor did not show a statistically significant association with mental health experiences, the majority of the adolescent respondents were black (more than 60%). So some consideration I believe is important in the topic of discussion.

**Authors’ response:** We appreciate the reviewer’s thoughtful observation regarding the relevance of engaging more critically with the variable of race/skin color in the context of our study. In response, we expanded this point by incorporating a critical reflection on the racial composition of our sample and its implications (page 31, lines 411-420). While race did not demonstrate a statistically significant association with the mental health outcomes in our adjusted models, we recognize that statistical insignificance does not equate to theorical irrelevance, particularly within the Brazilian context, where racialized structures continue to shape health disparities in multifaceted ways. Accordingly, we have included a discussion (page 31, lines 421-428) that situates the high proportion of Black adolescents in our sample within a broader sociohistorical framework, drawing from recent literature on the racialized impacts of the Covid-19 pandemic and the enduring effects of structural racism. This addition aims to draw attention to the analytical limitations of a strictly quantitative approach when addressing phenomena deeply embedded in systemic inequalities and to acknowledge race not merely as a variable, but as a constitutive dimension of lived experience and mental health vulnerability.

**Reviewer #2:**

Dear Reviewer 2,

Thank you for your insightful observations. Below, we provide detailed responses to each of the points you raised.

1. The background could benefit from a more focused review of existing studies on adolescent mental health during COVID-19, particularly in Latin America or similar socio-economic contexts, to better position the study’s contribution.

**Authors’ response:** We thank the reviewer’s suggestion to incorporate a more focused review of studies on adolescent mental health during Covid-19 in Latin America or comparable socio-economic settings. Given that the present study was conducted in Brazil, we deliberately chose to concentrate the contextual background on Brazilian research (page 4, lines 84-99). This decision was guided by the need to align the literature review with the specific socio-political and structural conditions shaping adolescents’ experiences in the country. Brazil’s stark social inequalities, regionally diverse pandemic responses, and unique public health infrastructure warrant a nationally grounded analysis. In light of these factors, this study seeks to reaffirm its empirical relevance and contextual coherence.

2. Regarding the sample, the use of a non-probabilistic sampling method limits generalizability, so it is important to clarify how participants were selected and to discuss potential biases.

**Authors’ response:** We acknowledge the inherent limitations of employing a non-probabilistic sampling method (pages 33-34, lines 476-479). In response, we have clarified the description of the sampling procedure within the Data Collection and Measures section (page 6, lines 137-146), specifying that participants were recruited during home visits conducted by trained health professionals in collaboration with Community Health Workers (CHWs) familiar with the local territory. Furthermore, we explicitly address the restricted generalizability of the results and critically examine the potential for selection bias, particularly the propensity to overrepresent households exhibiting stronger ties to primary healthcare services or greater accessibility to CHWs. These revisions serve to increase transparency concerning the methodological constraints inherent to the study design.

3. Providing details about demographic characteristics, such as socioeconomic status and ethnicity, would also help contextualize the findings.

**Authors’ response:** We appreciate the reviewer’s recommendation to include more detailed demographic characteristics to enhance the contextualization of our findings. In response, we expanded the Introduction (pages 4-5, lines 100-110) to provide a comprehensive description of the study setting and target population. This revised section underscores São Paulo’s demographic landscape, with particular attention to the concentration of adolescents in socioeconomically vulnerable urban areas, thereby offering a clearer backdrop against which the study's results can be interpreted. While direct measures of participants’ socioeconomic status were not available, race/skin color and other relevant sociodemographic indicators were already presented at the outset of the Results section (page 10, lines 240-243). These data showed that 63.1% of the sample identified as Black, 90.7% attended public schools, 86.4% had employed parents, and 21.5% reported insufficient financial resources to secure adequate nourishment during the pandemic.

4. Data collection took place between November and December 2021, after the initial peak of the pandemic; therefore, it would be useful to address how this timing may have influenced the mental health assessments compared to earlier phases.

**Authors’ response:** Indeed, conducting the survey between November and December 2021 may have influenced mental health indicators by attenuating the effects of acute stressors experienced during earlier phases. In our revision (page 34, lines 479-482), we clarified that this period constituted a transitional phase characterized by psychological readjustment, during which adolescents were contending with the aftermath of prolonged disruption, persistent uncertainty, and uneven recovery trajectories.

5. While the scales used in the study are well-described, the justification for the cutoff points, such as using GAD-7 ≥10 for moderate/severe anxiety, should be explicitly referenced, especially considering cultural validation.

**Authors’ response:** Thank you for raising this important point. In response, we have expanded the Data Collection and Measures subsection to include explicit justification and references for the cutoff points used for both the GAD-7 and PSS-10 scales. Specifically, for the GAD-7, we cited Spitzer et al. (2006), who originally proposed the ≥ 10 threshold based on high sensitivity and specificity in identifying generalized anxiety disorder. We also referenced Capellini et al. (2023), who applied this cutoff in a Brazilian sample, as well as the meta-analysis by Plummer et al. (2016), which supports its cross-cultural validity (page 7, lines 160-167). For the PSS-10, we referenced Wiriyakijja et al. (2020) to justify the ≥ 14 cutoff, acknowledging that although no culturally validated threshold exists for Brazilian adolescents, this categorization aligns with international conventions and has been used to capture clinically meaningful levels of perceived stress (pages 7-8, lines 176-182).

We also acknowledged that, while a culturally specific cutoff has not been formally established for Brazilian adolescents, our categorization remains consistent with the interpretive framework of the original scale. Following the approach adopted by the aforementioned authors, we combined the moderate and high stress categories to capture more pronounced levels of psychological distress. This strategy does not contravene validated thresholds; rather, it aligns with established methodological precedents intended to highlight more exacerbated effects across the assessed dimensions.

6. The statistical analysis section currently lacks a complete description of the multivariate modelling process, so the full methodology including criteria for variable inclusion or exclusion, handling of confounders, and checks for multicollinearity should be clearly detailed, and it would strengthen the paper to include model fit indices or measures of predictive validity.

**Authors’ response:** Thank you for this important observation. In response, we thoroughly revised the Statistical Analysis subsection of the Methods section to provide a clearer and more comprehensive account of our modelling strategy (page 9, lines 214-225).

We now specify that descriptive analyses were conducted using absolute and relative frequencies. To assess differences in proportions between girls and boys, Pearson’s chi-square test was applied. Subsequently, we employed binomial logistic regression to explore associations between explanatory variables (organized into thematic domains) and each outcome: moderate to severe anxiety and moderate to high perceived stress. Univariate logistic regressions were initially used to assess the independent association of each explanatory variable with the outcomes. Variables with p < 0.05 in the domain-specific multivariate models were considered eligible for inclusion in the final model. The final multivariate logistic regression models were built by including variables that (1) remained statistically significant in the multivariate analysis, (2) were identified as confounders — defined as producing a ≥ 10% change in the odds ratio when comparing crude and adjusted estimates — and/or (3) were retained based on theoretical relevance. We also report that multicollinearity was evaluated using the Variance Inflation Factor (VIF), and model fit was assessed using the Hosmer-Lemeshow goodness-of-fit test and Nagelkerke’s pseudo R^2^.

As part of these methodological clarifications and refinements, we reviewed and corrected the regression models presented in the tables, which had previously omitted confounder adjustments. The final models are now properly adjusted, and all variables included in the models are explicitly listed in the table footnotes and described in the Results section. In addition, we improved the legibility and clarity of all tables by updating their color scheme and reorganizing the footnotes. These corrections necessitated modifications to the Results (page 14, lines 226-281; pages 21-22, lines 295-319), as well as cascading updates to the Discussion (pages 29-30, lines 376-382; page 32, lines 429-441; page 33, lines 465-469) and Conclusion (page 36, lines 540-542) to ensure consistency with the revised analyses.

**New added references:**

[8] Cabral CS, Guimarães JS, Teixeira A, Generoso NK, França Junior I, Borges ALV. “We want to hug a friend”: the COVID-19 pandemic among low-income adolescents. Rev Saude Publica. 2023;57(Suppl 1):5s. doi: 10.11606/s1518-8787.2023057004778

[15] Mokhwelepa LW, Sumbane GO. Men’s Mental Health Matters: The Impact of Traditional Masculinity Norms on Men’s Willingness to Seek Mental Health Support; a Systematic Review of Literature. Am J Mens Health. 2025 May-Jun;19(3):15579883251321670. doi: 10.1177/15579883251321670

[19] Maruyama JM, Tovo-Rodrigues L, Santos IS, Murray J, Matijasevich A. Changes and predictors of adolescent emotion regulation, self-esteem, and locus of control during the COVID-19 pandemic: 2004 Pelotas Birth Cohort. Braz J Psychiatry. 2023 Sep-Oct;45(5):389-96. doi: 10.47626/1516-4446-2023-3169

[20] Barros MBA, Lima MG, Malta DC, Azevedo RCS, Fehlberg BK, Souza Júnior PRB, et al. Mental health of Brazilian adolescents during the COVID-19 pandemic. Psychiatry Res Commun. 2022;2:100015. doi: 10.1016/j.psycom.2021.100015

[21] IBGE. Censo Demográfico 2022. Rio de Janeiro: Instituto Brasileiro de Geografia e Estatística; 2022. Available from: https://biblioteca.ibge.gov.br/index.php/biblioteca-catalogo?view=detalhes&id=2101637

[29] Capellini VK, Paro FM, Vieira RD, Wittmer VL, Barbalho-Moulim MC, Soares SCS, Oliveira CGT, Duarte H. Brazilian physiotherapist anxiety and depression during the COVID-19 pandemic: a cross-sectional survey. Cien Saude Colet. 2023;28(10):2951-63. doi: 10.1590/1413-812320232810.09922023

[30] Plummer F, Manea L, Trepel D, McMillan D. Screening for anxiety disorders with the GAD-7 and GAD-2: a systematic review and diagnostic metaanalysis. Gen Hosp Psychiatry. 2016 Mar-Apr;39:24-31. doi: 10.1016/j.genhosppsych.2015.11.005

[32] Wiriyakijja P, Porter S, Fedele S, Hodgson T, McMillan R, Shephard M, Riordain RN. Validation of the HADS and PSS-10 and a cross-sectional study of psychological status in patients with recurrent aphthous stomatitis. J Oral Pathol Med. 2020;49:260-270. doi: 10.1111/jop.12991

[45] Plan International Brasil. Por ser menina no Brasil: crescendo entre direitos e violências – adolescentes participando da construção de um país mais justo para meninas. São Paulo: Plan International Brasil; 2021. 112 p. Available from: https://plan.org.br/wp-content/uploads/2021/11/relatorio-por-ser-menina-final.pdf

[47] Moura HSD, Canatto MCD, Berra TZ, Silva RVS, Rosa RJ, Campos MCT, Costa FBP, Ribeiro NM, Teibo TKA, Araújo JST, Tártaro AF, Alves YM, Souza RMP, Uchoa SAC, Ferezin LP, Arcêncio RA. Food insecurity, income loss, healthcare access, and other exacerbated social inequalities among people experiencing homelessness during the COVID-19 pandemic in Brazil (2021–2023). Discov Public Health. 2024;21(17):1-17. doi: 10.1186/s12982-024-00141-w

[48] Santos M, Penteado JO, Brum RL, Bonifácio AS, Ramires PF, Gariboti DF, Cardoso RMS, Júnior FMRS. Ethnic/Racial Disparity in Mortality from COVID-19: Data for the Year 2020 in Brazil. Spat Demogr. 2023;11(1):1-17. doi: 10.1007/s40980-022-00112-2

[49] van Loon AWG, Creemers HE, Vogelaar S, Saab N, Miers AC, Westenberg PM, Asscher JJ. Trajectories of adolescent perceived stress and symptoms of depression and anxiety during the COVID-19 pandemic. Sci Rep. 2022;12:15957. doi: 10.1038/s41598-022-20344-y

[55] Peterman A, O'Donnell M, Potts A. COVID-19 and violence against women and children: a second research round up. Center for Global Development; 2020. Available from: https://www.cgdev.org/publication/covid-19-and-violence-against-women-and-children-second-research-round

[56] Van Gelder N, Peterman A, Potts A, O'Donnell M, Thompson K, Shah N, et al. COVID-19: reducing the risk of infection might increase the risk of intimate partner violence. eClinicalMedicine. 2020;21:100348. doi: 10.1016/j.eclinm.2020.100348

[57] Roesch E, Amin A, Gupta J, García-Moreno C, Ellsberg M. Violence against women and girls during COVID-19 pandemic: emerging evidence of the shadow pandemic. Lancet. 2020;395(10242):657-8. doi: 10.1016/S0140-6736(20)30523-X

[58] Gonçalves FS, Machado DF. Violência contra as crianças durante a covid-19: aprendizagens deixadas pela pandemia. Serv Soc Soc. 2025;148(1):e-6628427. doi: 10.1590/0101-6628.427

[59] Marques ES, Moraes CL, Hasselmann MH, Deslandes SF, Reichenheim ME. Violence against women, children, and adolescents during the COVID-19 pandemic: overview, contributing factors, and mitigating measures. Cad Saúde Pública. 2020;36(4):e00074420. doi: 10.1590/0102-311X00074420

[60] Campos MCT, Rosa RJ, Ferezin LP, Berra TZ, Moura HSD, Tartaro AF, Alves YM, Tavares RBV, Ribeiro NM, Araújo JST, Costa FBP, Fiorati RC, Serrano-Gallardo MDP, Arcêncio RA. Violence experienced by women, their mental health status, and determinants in favelas under the pandemic COVID-19 in Brazil. BMC Womens Health. 2025;25:242. doi: 10.1186/s12905-025-03793-1

[61] Rodrigues C, Leitão M, Barros RD. Interseccionalidade e violência contra as mulheres em tempos de pandemia de covid‑19: diálogos e possibilidades. Saúde Soc. 2021;30(2):e200367. doi: 10.1590/S0104-12902021200367

**The following two references were excluded from the original list:**

Fiocruz. Jovens e saúde: revelações da pandemia no Brasil 2020–2022. Sobrinho ALS, Abramo HW, Villi MC, editors. 23rd ed. Rio de Janeiro: Fiocruz; 2022.

Ramaiya A, Mmari K, Borges AL, Cabral CS, Mafuta E, Aimee L, Yu C, Pinandari AW, Wilopo SA, Chipeta E, Hunersen K. Understanding how socioecological factors affect COVID-19 vaccine perceptions among adolescents: qualitative evidence from seven high-, middle- and low-income countries. JoGHR. 2024;8:e2024009. doi: 10.29392/001c.94937.
